# No diurnal variation of classical and candidate biomarkers of Alzheimer’s disease in CSF

**DOI:** 10.1186/s13024-016-0130-3

**Published:** 2016-09-07

**Authors:** Claudia Cicognola, Davide Chiasserini, Paolo Eusebi, Ulf Andreasson, Hugo Vanderstichele, Henrik Zetterberg, Lucilla Parnetti, Kaj Blennow

**Affiliations:** 1 Institute of Neuroscience and Physiology, Department of Psychiatry and Neurochemistry, The Sahlgrenska Academy at University of Gothenburg, House V3, SU / Mölndal hospital, Göteborgsvägen 31, SE-431 80 Mölndal, Sweden; 2 Clinical Neurochemistry Laboratory, Sahlgrenska University Hospital, SE-431 80 Mölndal, Sweden; 3 Section of Neurology, Department of Medicine, Center for Memory Disturbances, University of Perugia, Sant’ Andrea delle Fratte, 06132 Perugia, Italy; 4 ADx NeuroSciences, Gent, Belgium; 5 UCL Institute of Neurology, Queen Square, London, UK

## Abstract

**Background:**

Cerebrospinal fluid (CSF) biomarkers have gained increasing importance in the diagnostic work-up of Alzheimer’s disease (AD). The core CSF biomarkers related to AD pathology (Aβ42, t-tau and p-tau) are currently used in CSF diagnostics, while candidate markers of amyloid metabolism (Aβ38, Aβ40, sAPPα, sAPPβ), synaptic loss (neurogranin), neuroinflammation (YKL-40), neuronal damage (VILIP-1) and genetic risk (apolipoprotein E) are undergoing evaluation. Diurnal fluctuation in the concentration of CSF biomarkers has been reported and may represent a preanalytical confounding factor in the laboratory diagnosis of AD. The aim of the present study was to investigate the diurnal variability of classical and candidate CSF biomarkers in a cohort of neurosurgical patients carrying a CSF drainage.

**Method:**

Samples were collected from a cohort of 13 neurosurgical patients from either ventricular (*n* = 6) or lumbar (*n* = 7) CSF drainage at six time points during the day, 1–7 days following the neurosurgical intervention. Concentrations of the core biomarkers were determined by immunoassays.

**Results:**

Although absolute values largely varied among subjects, none of the biomarkers showed significant diurnal variation. Site of drainage (lumbar vs. ventricular) did not influence this result. The different immunoassays used for tau and Aβ markers provided similar results.

**Conclusion:**

Time of day at CSF collection does not ultimately affect the concentration levels of classical and candidate AD biomarkers. Similar trends were found when using different immunoassays, thus corroborating the consistency of the data.

**Electronic supplementary material:**

The online version of this article (doi:10.1186/s13024-016-0130-3) contains supplementary material, which is available to authorized users.

## Background

Alzheimer’s disease (AD) represents the most common neurodegenerative disease leading to dementia, and its prevalence is increasing and becoming a major health and socioeconomic issue [1]. AD pathology affects the brain several years before the clinical onset, which is characterized by a long asymptomatic phase followed by a prodromal phase with disturbances in episodic memory, ultimately leading to overt dementia [2]. To enable early initiation of treatment, especially the day disease-modifying drugs reach the clinic, early diagnosis is recommended, which is based on combining clinical symptoms and neuropsychological testing with biomarkers (CSF, imaging) reflecting AD pathology [3]. With respect to classical CSF biomarkers (Aβ42, total tau, phosphorylated tau), large evidence has been collected about their reliability in supporting the diagnosis of AD [4–6].

In order to introduce the use of biomarkers as part of the routine diagnostic assessment, standardization of the procedures is mandatory. Preanalytical factors are reported to be the cause of at least 40–60 % of the total variability in biomarker measurements [7]. Some key issues were identified for sample collection and analysis, such as presence of a CSF gradient, blood contamination of the sample, fasting state of the patient, and other laboratory procedures regarding collection and storage of the samples (collection tubes, aliquot volume, centrifugation, length and temperature of storage, number of freeze/thaw cycles).

The major focus of our study is the influence of time of day at CSF collection which, in a clinical routine schedule, may influence the results obtained. If proved significant, the diurnal fluctuations would affect the reliability of the CSF analysis depending on time at withdrawal. Only few studies have addressed this issue, giving inconsistent results: Bateman et al. first observed a significant decreasing trend of Aβ during the 24 h in young individuals [8], an observation disproved in subsequent studies in older populations [9–11], the ones more likely to undergo CSF analysis to assess the risk of memory impairment and AD. There is also no consensus on the time intervals between the CSF withdrawals or the volume to be taken.

Regarding analytical factors, different methods and assays may also represent another source of variability [12], but no study has been done on the performance and reliability of different assays when considering diurnal variation as a confounding factor.

### Candidate biomarkers

Along with core biomarkers of AD, other proteins are currently studied as candidate biomarkers, with significant results in their role in CSF diagnostics.

Biomarkers of the amyloidogenic pathway are being taken into account in AD diagnosis, since Aβ42 is produced from cleavage of amyloid precursor protein (APP). APP can be cleaved either by α- or β-secretase, releasing sAPPα or sAPPβ, respectively. After β-secretase cleavage, the remaining C-terminal fragment is further processed by γ-secretase which releases Aβ42 along with Aβ38 and Aβ40 fragments, whereas no amyloidogenic fragments are produced after α-secretase cleavage followed by γ-secretase. Since sAPPα was found decreased and sAPPβ increased in AD subjects, α- and β- pathway were considered mutually exclusive [13–16]; however, subsequent studies showed positively correlated concentrations of sAPPα and β [17–19], challenging the hypothesis of an imbalance between the two isoforms. The several critical steps of amyloid metabolism can also cause Aβ fluctuations during the day: Dobrowolska et al. found diurnal fluctuations in sAPPα, sAPPβ, Aβ40, and Aβ42, diminishing with increased age [20].

Regarding genetic risk factors for AD, ApoE is probably the most important and acknowledged. The three different isoforms exert a different effect on AD predisposition: ApoE4 increases the risk of AD, whereas ApoE2 is a protective factor, compared to the most common variant ApoE3. Quantifying ApoE isoforms, especially ApoE4, could be a useful biological correlate in the study of AD pathology, but CNS and peripheral ApoE isoform turnover rates differ substantially, probably because the ApoE metabolism pathways are different in the central nervous system (CNS) and the periphery, as observed in a study by Wildsmith et al. [21]. Time-dependent fluctuations were also observed [21, 22].

Also neurogranin (a calmodulin-binding postsynaptic protein, involved in synaptic signaling, plasticity, long-term potentiation and memory consolidation) is currently studied as a potential biomarker. Synaptic loss has been reported to occur very early in the natural history of AD, therefore neurogranin can be a valuable biomarker of early, possible preclinical, stage of the disease [23]. Neurogranin concentrations are significantly increased in mild cognitive impairment (MCI) [24, 25] and can be a predictive factor of conversion to dementia.

Among inflammatory markers we find YKL-40 (also known as chitinase-3-like protein 1), a protein mainly expressed by astrocytes. Even if its physiological function remains unclear [26], it was found to be upregulated in the AD brain from the preclinical stages of the disease and may have a role in Aβ deposition [27]. Besides, as a biomarker, it could also give additional information on the inflammatory status of the AD brain. However, studies on its diagnostic and prognostic value still give conflicting results [26, 28].

Neuronal damage markers include VILIP-1 (visinin-like protein 1), a neuronal calcium sensor protein involved in calcium-mediated neurotoxicity. Not only VILIP-1 is significantly increased in AD and could help differentiate AD from other dementia, but it may also have an influence on tau metabolism [29, 30]. Moreover, when combined with Aβ42, it is also a good predictor of cognitive decline [31, 32].

The diurnal variability of the aforementioned biomarkers has been poorly investigated and no studies are available on neurogranin, YKL-40 and VILIP-1. Also, most studies only use one immunoassay type and do not provide a comparison between different methods.

### Aim

In this study we wanted to measure classical (Aβ42, t-tau, p-tau) and candidate (Aβ38, Aβ40, sAPPα, sAPPβ, apolipoprotein E, neurogranin, YKL-40 and VILIP-1) AD biomarkers in CSF collected at six time points (08:00; 12:00; 16:00; 20:00, 00:00, 08:00) during 24 h to assess these markers for diurnal variation.

## Methods

### Participants

We enrolled a total of 13 patients, 8 males (61.5 %) and 5 females (38.5 %), with an age span between 26 and 82 years (Tables 1 and 2). All patients carried a CSF drainage after neurosurgical intervention for tumors, traumas or hemorrhages (*n* = 9) or for monitoring CSF pressure (*n* = 4). 7 patients had a lumbar drainage (53.8 %) and 6 patients had a ventricular drainage (46.2 %), with no filter (Codman&Shurtleff, Inc, Raynham, MA, USA). CSF was collected according to a standard protocol following international guidelines [33]. The study was approved by the local Ethical Committee and informed written consent was signed by all patients enrolled. The work was carried out according to the Declaration of Helsinki.

**Table 1:** Age, clinical features of the patients and type of drainage

Patient	Age	Case of drainage placement	Type of drainage
1	48	Third ventricle tumor	ventricular
2	73	Left middle cerebral artery infarct	ventricular
3	81	Subarachnoid hemorrhage	ventricular
4	72	Intracranial hemorrhage	lumbar
5	67	Cerebellar hemorrhage	ventricular
6	69	Cerebellopontine angle tumor	lumbar
7	74	Normal pressure hydrocephalus	lumbar
8	36	Head trauma	ventricular
9	63	Intracranial hemorrhage	ventricular
10	58	Tetraventricular hydrocephalus	lumbar
11	77	Normal pressure hydrocephalus	lumbar
12	82	Normal pressure hydrocephalus	lumbar
13	26	Pseudotumor cerebri	lumbar

**Table 2:** Demographics (mean ± standard deviation or count with percentages)

Age	63.5 ± 17.3
Sex	Male: 8 (61.5 %)
	Female: 5 (38.5 %)
Type of drainage	Lumbar: 7 (53.8 %)
	Ventricular: 6 (46.2 %)
Cause of drainage placement	Cerebellar hemorrhage: 1 (7.7 %)
	Cerebellopontine angle tumor: 1 (7.7 %)
	Head trauma: 1 (7.7 %)
	Intracranial hemorrhage: 2 (15.4 %)
	Left middle cerebral artery infarct: 1 (7.7 %)
	Normal pressure hydrocephalus: 3 (23.1 %)
	Pseudotumor cerebri: 1 (7.7 %)
	Subarachnoid hemorrhage: 1 (7.7 %)
	Tetraventricular hydrocephalus: 1 (7.7 %)
	Third ventricle tumor: 1 (7.7 %)

### CSF collection

According to our protocol, 4 mL CSF were collected at six time points: 8.00, 12.00, 16.00, 20.00, 00.00 and 8.00. Haemorrhagic CSF samples were excluded; if the drainage was put after a trauma, haemorrhage or tumor surgery CSF samples collected had to be either clear or slightly and stably xantochromic (<1000 rbc/μL). CSF was collected in polypropylene (PP) tubes (Sarsted, code: 62.610.201) and all samples were centrifuged (2000 g × 10 min, room temperature) no longer than 15 min after collection, aliquoted in 0.5 ml aliquots (Sarsted, code: 72.730.007)﻿ and frozen at −80 °C pending analysis (Table 3).

**Table 3:** CSF collection procedures and sample processing

Subjects:	13 neurosurgical patients carrying a CSF drainage
	8 M, 5 F; 26 to 82 y
	Admitted for tumor, trauma, hemorrhage (*n* = 9), CSF pressure monitoring (*n* = 4)
CSF collection:	From lumbar (*n* = 7) or ventricular (*n* = 6) drainage
	Volume: 4 mL in PP Sarsted tubes, code: 62.610.201
	Time of day: 8.00, 12.00, 16.00, 20.00, 00.00, 8.00
CSF processing:	Centrifugation within 15 min, 2000 g × 10 min at RT
	0.5 mL aliquots in PP Sarsted tubes, code: 72.730.007
CSF storage:	−80 °C freezer, with controlled temperature, alarm and CO2 backup system
Thawing:	RT with gentle shaking
Biomarker measurement:	Immunoassay (Fujirebio): Aβ1-42, t-tau, p-tau181
	Immunoassay (Biovendor): VILIP-1
	Immunoassay (R&D): YKL-40
	Immunoassay (Euroimmun): Aβ1-38, Aβ1-40, Aβ1-42, t-tau, ApoE
	Immunoassay (in-house): neurogranin
	Immunoassay (MSD): Aβx-38, Aβx-40, Aβx-42, sAPPα, sAPPβ

### Assays

Aβ42, t-tau and p-tau were determined with ELISA kit (Fujirebio) in the Clinical Neurochemistry Laboratory of the University of Perugia (Perugia, Italy), according to the manufacturer’s protocol.

Aβ38, Aβ 40, Aβ 42, sAPPα, sAPPβ, neurogranin, VILIP-1 and YKL-40 were analyzed in the Clinical Neurochemistry Laboratory of The Sahlgrenska Academy, University of Gothenburg (Mölndal, Sweden).

MSD V-PLEX Plus kits were used for Aβ38, Aβ40, Aβ42, sAPPα and sAPPβ, according to the manufacturer’s protocol. ELISA kits from R&D and Biovendor were used for YKL-40 and VILIP-1, respectively, according to the manufacturer’s protocol.

An in-house developed assay was used for neurogranin, according to previous reports [24]. Briefly, in-house antibody Ng7 diluted in PBS was used as coating, a rabbit anti-neurogranin antibody (ab23570, cat. no. 07-425, Upstate, Lake Placid, NY, USA) as primary and a goat anti-rabbit sulfo-tag antibody (MSD) for detection. Full-length Ng 1–78 protein (ADx) was used as calibrator for the standard curve.

Aβ38, Aβ40, Aβ42, t-tau, and ApoE were also measured with Euroimmun kits at ADx NeuroSciences (Gent, Belgium).

Together with the quality control (QC) samples included in the kits, CSF pools were run in each plate as further internal controls to check for variability, as previously described [34]. Performance data (inter- and intra assay variability, recovery and linearity) can be found at the following references [34, 35].

### Statistical analysis

Statistical analysis was performed using R v 13.1 and Graph Pad v 6.0. Continuous variables were reported as means, standard deviations and ranges. Categorical variables were shown as counts and percentages. Continuous variables were tested for normality with Shapiro–Wilk test. Differences in biomarkers levels across time points were tested by means of non-parametric version of repeated measurement ANOVA based on aligned ranks, due to the non-normality of the majority of parameters. Spearman’s r correlation coefficients of biomarkers concentrations were calculated pooling all the time points. Drainage type was tested as potential confounder in the fluctuations of biomarkers across time points. Bland-Altman plots were drawn to investigate the agreement of different assays for some of the investigated biomarkers (Aβ38, Aβ40, Aβ42, t-tau). *P* < 0.05 was chosen as the minimum level of statistical significance.

## Results

### Circadian trends in the individual classical and candidate biomarkers

**Fig. 1:**
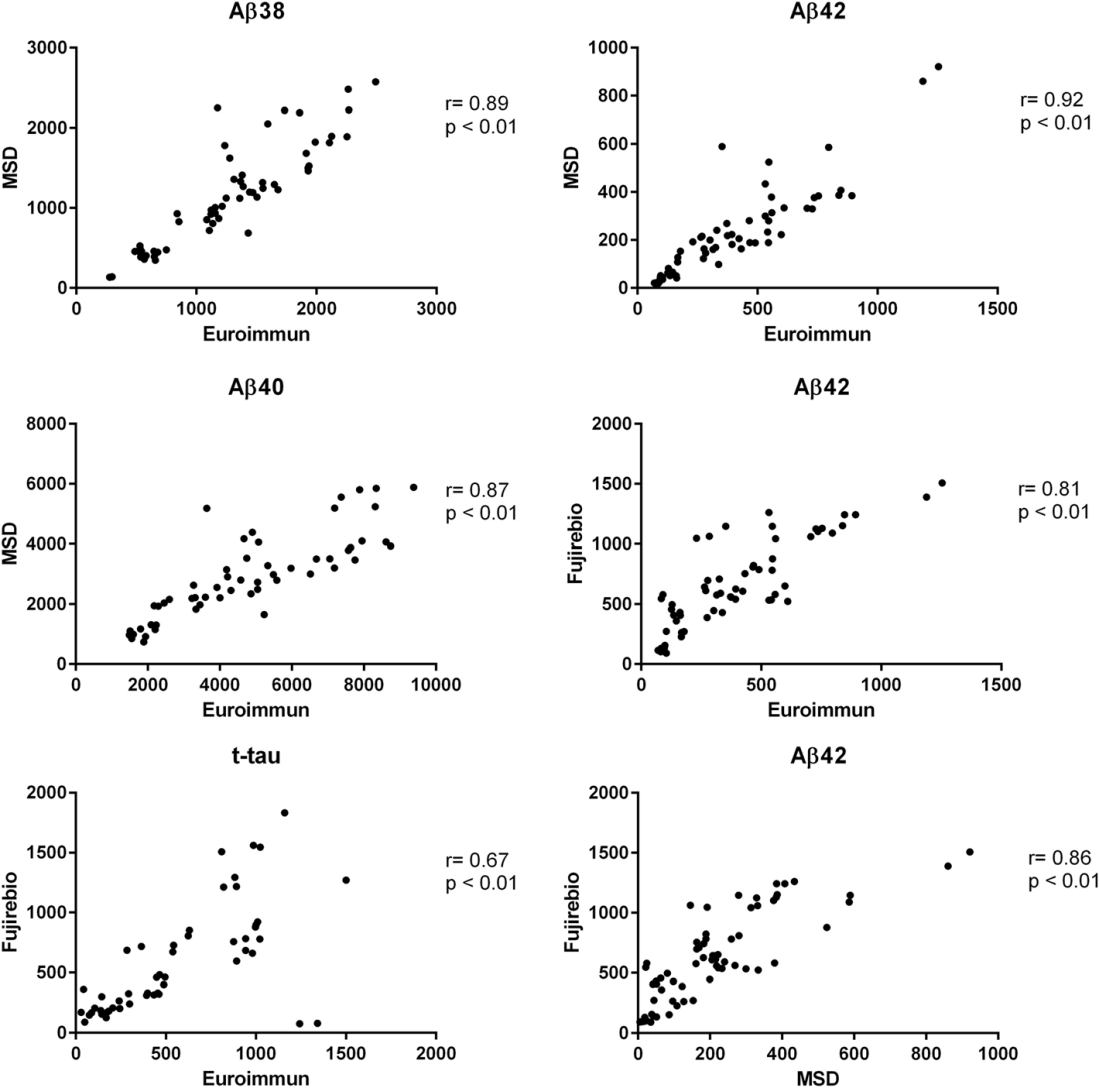
Correlation of the levels of biomarkers measured with different assays (Euroimmun, MSD, Fujirebio)

Aβ38, Aβ40, Aβ42 and t-tau measured with different methods, i.e. immunoassay by Euroimmun, Fujirebio, and MSD, showed significant positive correlations (Fig. 1). Results from Euroimmun vs. MSD highly correlated for both Aβ38 (*r* = 0.89, *p* < 0.01, 95 % confidence interval (CI) = 0.82 to 0.94) and Aβ40 (*r* = 0.87, *p* < 0.01, 95 % CI = 0.74 to 0.9). Similar results were found for Aβ42 when comparing Euroimmun vs. MSD (*r* = 0.92, *p* < 0.01, 95 % CI = 0.82 to 0.93), Euroimmun vs. Fujirebio (*r* = 0.81, *p* < 0.01, 95 % CI = 0.74 to 0.9) and MSD vs. Fujirebio (*r* = 0.86, *p* < 0.01, 95 % CI = 0.74 to 0.89) (Fig. 1).

There was also a positive correlation for tau when measured with Fujirebio and Euroimmun (*r* = 0.67, *p* < 0.01, 95 % CI = 0.48 to 0.8). Bland-Altman analysis was used to assess the agreement among the different assays for measuring absolute concentrations of the biomarkers (Additional file 1).

Notably, MSD assays overall showed differences either when compared to Euroimmun or Fujirebio assays. This was especially evident for Aβ42 and Aβ40 which showed the largest variation in the difference between the two assays across the whole range of concentrations (Additional file 1).

As expected from the different admittance diagnosis, sex and age of the population (Tables 1 and 2), absolute concentrations of the biomarkers were very different among patients (Table 4). For what concerns classical biomarkers, in the whole population Aβ42 ranged from 71 to 1253.4 pg/mL, t-tau from 29 to 1501 pg/mL and p-tau from 15 to 113 pg/mL.

**Table 4:** Concentration range of the biomarkers (with median)

Biomarker	Median (IQR)	Min - Max
Aβ_38_ MSD (pg/mL)	990.9 (440.1–1396.0)	33.3–2573.0
Aβ_40_ MSD (pg/mL)	2514.9 (1154.8–3356.5)	79.3–5880.5
Aβ_42_ MSD (pg/mL)	182.1 (54.1–266.6)	5.5–921.2
Aβ_38_ Euroimmun (pg/mL)	1247.7 (1120.9–1462.0)	277.5–2492.5
Aβ_40_ Euroimmun (pg/mL)	4669.0 (3605.0–5305.0)	1487.0–9382.2
Aβ_42_ Euroimmun (pg/mL)	337.9 (191.9–521.8)	70.6–1253.4
t-tau Euroimmun (pg/mL)	464.7 (223.0–917.6)	29.3–1501.0
Aβ_42_ Fujirebio (pg/mL)	568.0 (294.5–786.0)	91.0–1507.0
t-tau Fujirebio (pg/mL)	319.0 (180.2–742.2)	72.0–1832.0
p-tau (pg/mL)	43.0 (30.3–52.8)	15.0–113.0
sAPPα (ng/mL)	60.4 (32.5–178.8)	4.3–429.6
sAPPβ (ng/mL)	66.4 (25.5–175.0)	1.4–313.6
YKL-40 (ng/mL)	264.6 (151.9–348.4)	65.4–1020.0
VILIP-1 (pg/mL)	113.5 (99.0–135.0)	72.0–781.0
Neurogranin (pg/mL)	354.5 (275.9–435.4)	46.3–1291.1
ApoE (ng/mL)	24.5 (18.8–44.4)	3.4–104.2

**Fig. 2:**
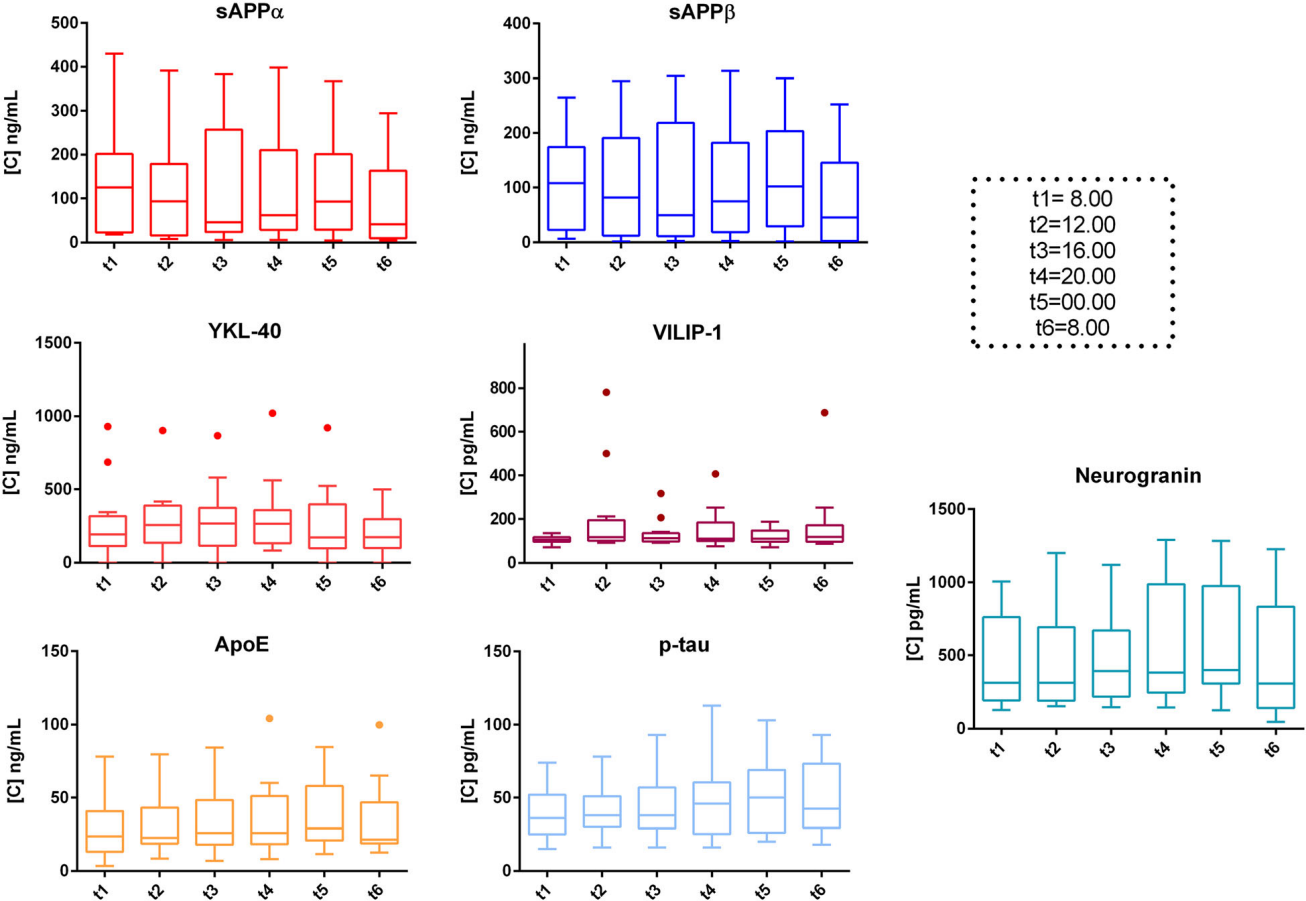
Concentration levels of the biomarkers across time points (horizontal lines representing the median, box representing the 25^th^ and 75^th^ percentiles, whiskers representing the 5^th^ and 95^th^ percentiles, and outliers represented by dots)

**Fig. 3:**
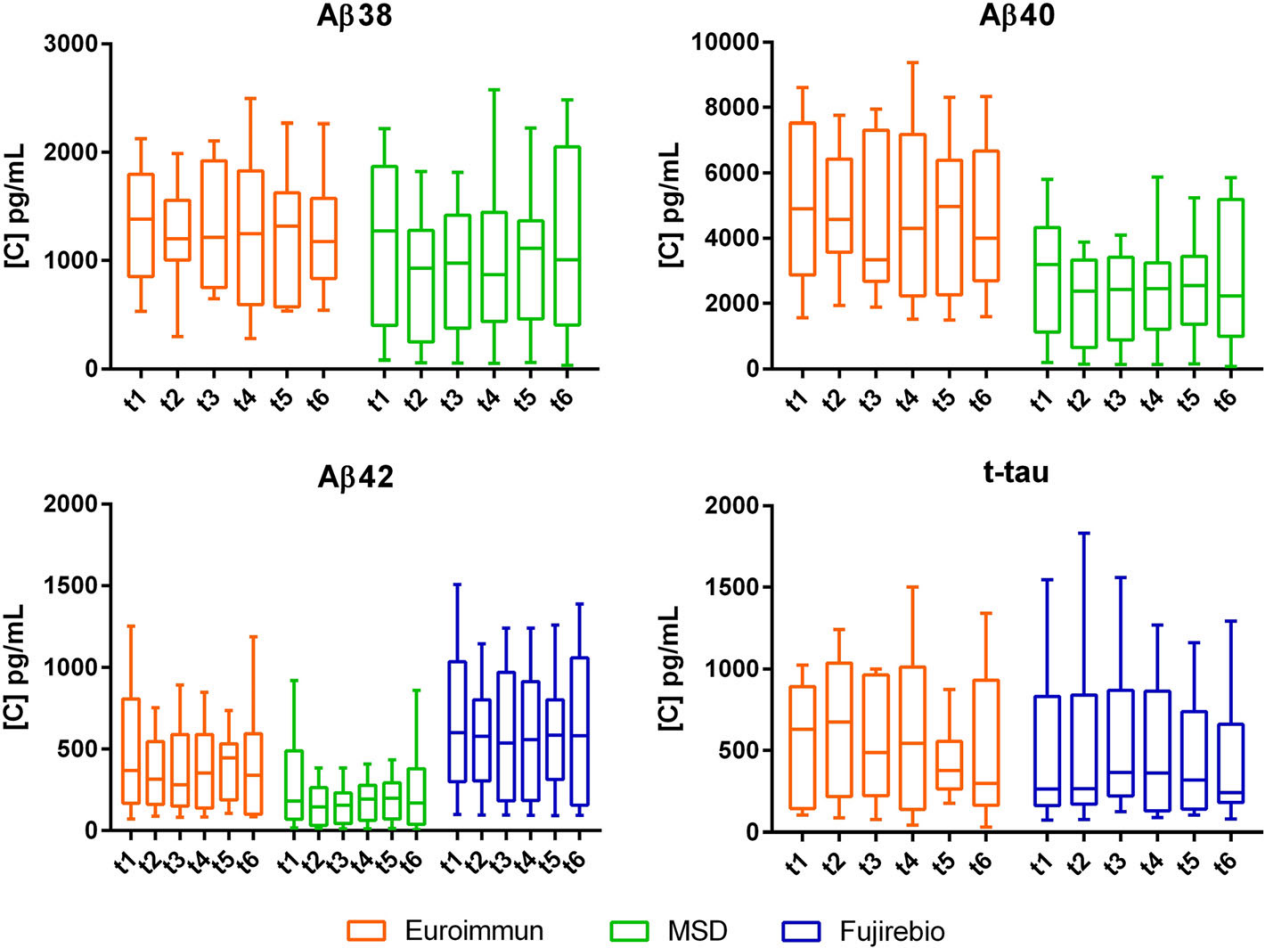
Concentration levels of several biomarkers measured with different assays (Euroimmun, MSD, Fujirebio) across time points (horizontal lines representing the median, box representing the 25^th^ and 75^th^ percentiles, whiskers representing the 5^th^ and 95^th^ percentiles)

Possibly due to their common metabolism pathway, amyloid metabolism markers (Aβ38, Aβ40, Aβ42, sAPPα, sAPPβ) showed similar trends over time in the whole population (Figs. 2 and 3). However, none of them had significant variation across time points (*p* > 0.05). sAPPα, sAPPβ and β-amyloid fragments correlated positively with each other, with r values ranging from to 0.54 to 0.97 (*p* > 0.01) (Additional file 2). When measured with Euroimmun kits, Aβ42/β40 and Aβ38/40 ratio showed no significant oscillations (*p* > 0.05). No significant intraindividual fluctuations over time were found for any of the Aβ markers when measured with the different assays (for Aβ42, see Additional file 3).

Neither T-tau nor P-tau did show a significant diurnal variation (Figs. 2 and 3). No significant oscillations were observed for Neurogranin, ApoE, YKL-40 and VILIP-1 (*p* > 0.05) (Fig. 2). Single patients showed some variations for specific proteins but globally no significant fluctuations over time were observed.

### Site of drainage

**Fig. 4:**
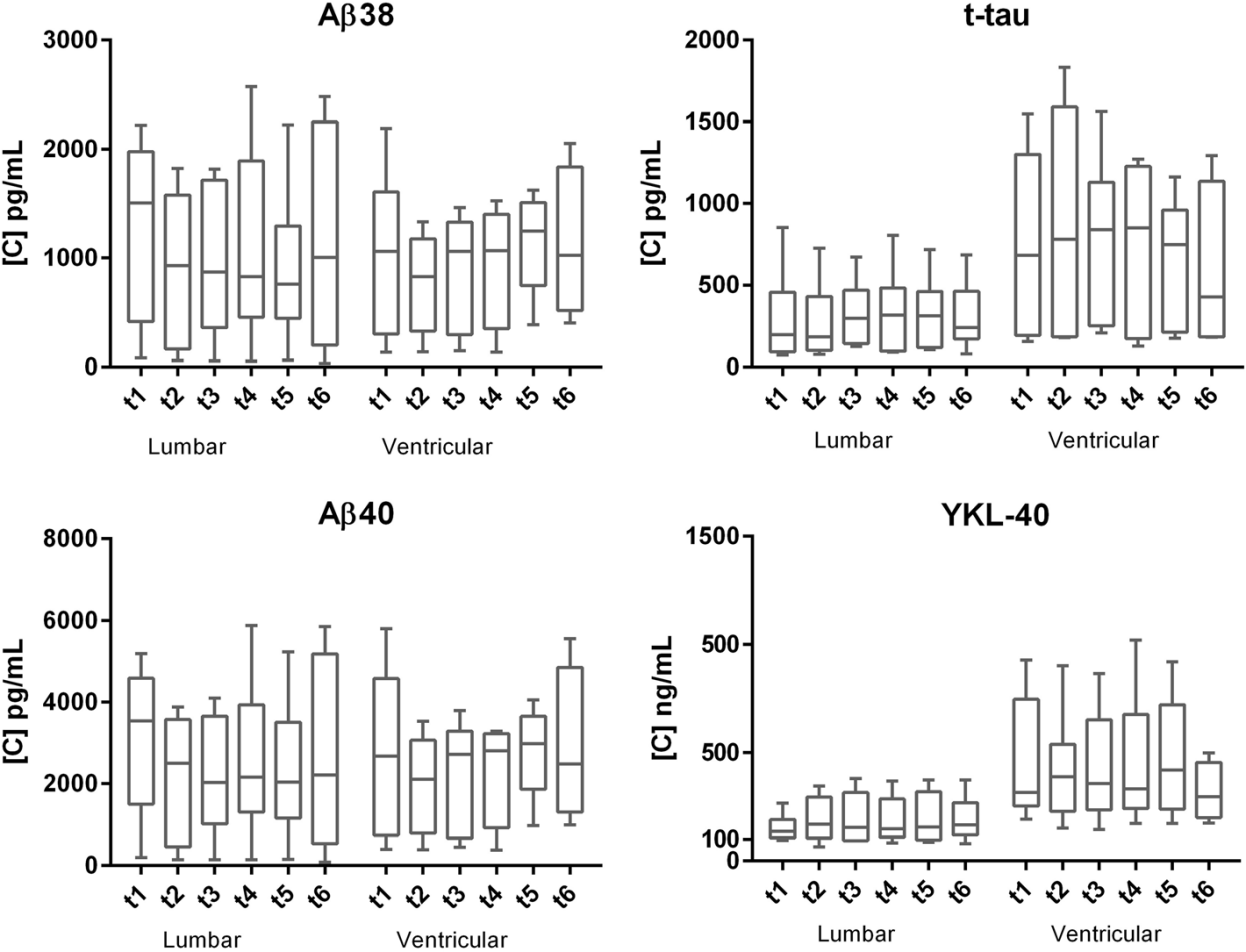
Concentration levels of several biomarkers with different type of drainage (lumbar or ventricular) across time points (horizontal lines representing the median, box representing the 25^th^ and 75^th^ percentiles, whiskers representing the 5^th^ and 95^th^ percentiles). Pictures not showed for non-significant comparisons

When site of drainage was considered as a covariate in non-parametric repeated measurements ANOVA based on aligned ranks, variability in some of the proteins (Aβ38, Aβ40, YKL40, t-tau) was found to be significantly explained by the site of drainage (*p* < 0.05), with generally higher concentration in CSF from ventricular catheter. Anyway, subgroup analyses restricted to lumbar or ventricular catheter showed no significant changes across the time points analysed (*p* > 0.05) (Fig. 4). Similar results were obtained when the analysis was repeated considering only patients with normal pressure hydrocephalus (*n* = 3, *p* > 0.05).

## Discussion

In our study we assessed the possible circadian oscillation of classical (Aβ42, t-tau, p-tau) and candidate (Aβ38, Aβ40, sAPPα, sAPPβ, apolipoprotein E, neurogranin, YKL-40, VILIP-1) CSF AD biomarkers. Previous studies addressed the issue of diurnal fluctuations of Aβ and tau in CSF (for review see [36]), however, a significant circadian oscillation of Aβ was detected only in the study by Bateman and colleagues [8]. Moreover, the study included subjects significantly younger than the ones who usually undergo lumbar puncture as a diagnostic procedure (23 to 78 years old).

Subsequent studies did not observe the same trend [9–11]. Bjerke et al. led a study in healthy subjects undergoing knee surgery and observed a slight decrease in Aβ42 levels, which tended to return to baseline after 24 h [10]. Slats et al. compared the trends of Aβ in healthy and mild AD subjects, noticing no significant diurnal variation and also a less pronounced circadian pattern compared with the one in younger subjects (59 to 85 years old) [11]. Only Moghekar et al. examined also tau along with Aβ, in a cohort of normal pressure hydrocephalus subjects, but no diurnal fluctuation of the biomarkers was reported [9].

Our study is in line with the majority of the results from previous ones, but we also investigated the possible oscillations of the latest candidate AD biomarkers (neurogranin, YKL-40, VILIP-1). We collected a smaller volume of CSF (4 mL) than in previous studies (from 6 to 40 mL) to avoid possible fluctuations due to tapping large volumes of CSF, and we compared results from ventricular CSF and lumbar CSF to detect a possible gradient effect.

Although CSF protein concentrations were significantly different from one patient to another, none of the biomarkers showed a significant diurnal variation. The ranges of the biomarkers were very different from the ones available from the literature on optimal cut-offs for AD biomarkers [37]. This is mostly due to the heterogeneity of the population, different for sex, age and diagnosis at admission (Tables 1 and 2). The subjects did all undergo an invasive procedure for catheter insertion, which might have affected the levels of the biomarkers in CSF. Moreover, 9 out of 13 subjects underwent brain surgery for tumours, haemorrhage or head trauma prior to drainage placement, which is most likely the reason for the generally upregulated values of inflammatory and brain injury markers (tau, YKL-40) in the population. Not to be excluded is also the presence of a possible underlying AD pathology in the older subjects. It is remarkable, however, that even if set on different ranges between subjects, the overall levels of the individual biomarkers are very stable over time and do not seem to be affected by external factors.

Site of drainage significantly influenced the variability of some of the biomarkers (Aβ38, Aβ40, YKL40, t-tau), as previously described [38], with generally higher concentration in CSF from ventricular catheter, most likely due to the increased concentrations of injury markers in the site of the surgical intervention or brain damage in patients carrying a ventricular drainage.

Some of the analytes tested in this work were measured with different immunoassays (Fujirebio, MSD, and Euroimmun). Absolute concentrations of the biomarkers using different immunoassays varied widely, possibly due to the different sources for the calibrators used by the manufacturers, as well as to the different antibodies used for coating and detection. No previous studies on circadian rhythms of CSF biomarkers have been performed comparing these three different techniques, which nevertheless showed good inter-assay correlations, proving the reliability of the methods in use and supporting the lack of circadian oscillations independently from the technique.

The main limitation of this study was the population, mostly admitted to hospital for traumatic intervention, limited in number and very different for age and disease. However, patients with normal pressure hydrocephalus, which were admitted to hospital only for diagnostic purposes and represented a more selected population, did not show significant diurnal variability as well. Obviously, to find ventricular CSF samples suitable for biomarker analysis is not easily manageable, due to the severe underlying conditions that lead to the insertion of a ventricular catheter. Same is for lumbar CSF: not being possible to perform multiple lumbar punctures during the day, repeated measurements at different time points can only be done after insertion of a drainage, which is seen as a dangerous procedure by most of the population, making it problematic to enrol healthy controls and AD subjects in larger studies. Further efforts are needed to raise public awareness about the minor risks of this kind of procedure, especially in view of an early diagnosis of AD pathology.

## Conclusions

In view of standardizing CSF collection and analysis procedures, the absence of an influence on classical and candidate CSF biomarker of time at sampling make them a valuable asset in the diagnostic work-up of AD. Independence from time of day is also crucial for clinical practitioners: being bound to fixed hospital schedules, the possibility to perform the lumbar puncture at any time of the day is of great importance.

An increasing number of tools are already being developed for the measurement of these analytes in CSF and preliminary results show robustness and significant overlap between different methods, proving their reliability and reproducibility for biomarker analysis. However, larger and selected populations are needed to increase the power of these findings.

## Additional files

Additional file 1: Bland-Altman plot for investigating the agreement between assays (mean difference and 95 % limits of agreement). (JPG 2632 kb) Additional file 2: Scatterplot and Spearman correlation coefficients with *p*-values for amyloid metabolism biomarkers measured with the three different assays. (JPG 1903 kb) Additional file 3: Intraindividual concentration levels of Aβ42 across time points measured with different assays (Euroimmun, MSD, Fujirebio). (JPG 2223 kb) Additional file 4: Raw data. (XLSX 28 kb)
