# Supplementary figures and images for: No diurnal variation of classical and candidate biomarkers of Alzheimer’s disease in CSF
Source: Mol Neurodegener. 2016 Sep 7;11(1):65. doi: 10.1186/s13024-016-0130-3 (PMC5013624; doi:10.1186/s13024-016-0130-3)

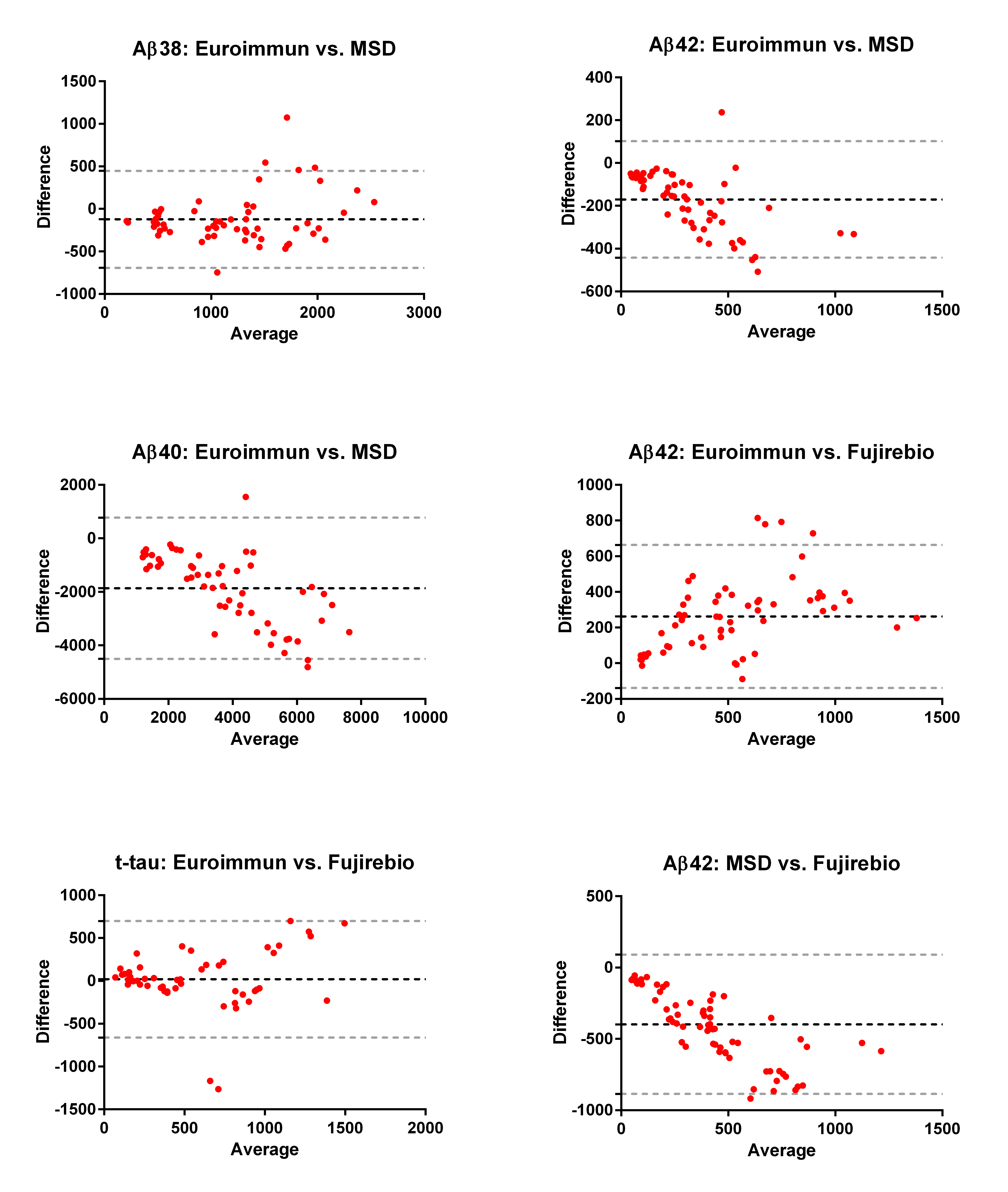

Supplement: Additional file 1: — Bland-Altman plot for investigating the agreement between assays (mean difference and 95 % limits of agreement). (JPG 2632 kb) [file 13024_2016_130_MOESM1_ESM.jpg]

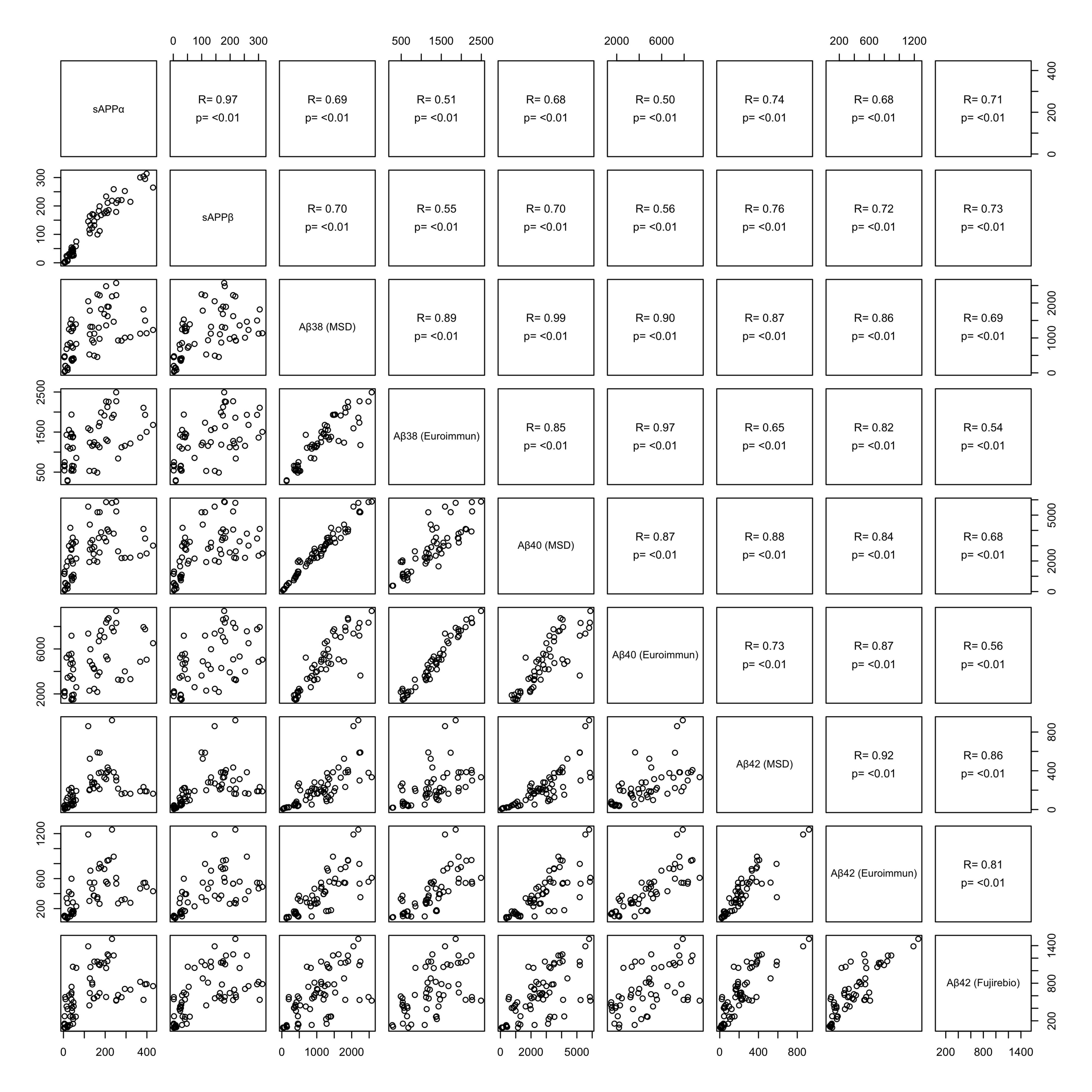

Supplement: Additional file 2: — Scatterplot and Spearman correlation coefficients with p-values for amyloid metabolism biomarkers measured with the three different assays. (JPG 1903 kb) [file 13024_2016_130_MOESM2_ESM.jpg]

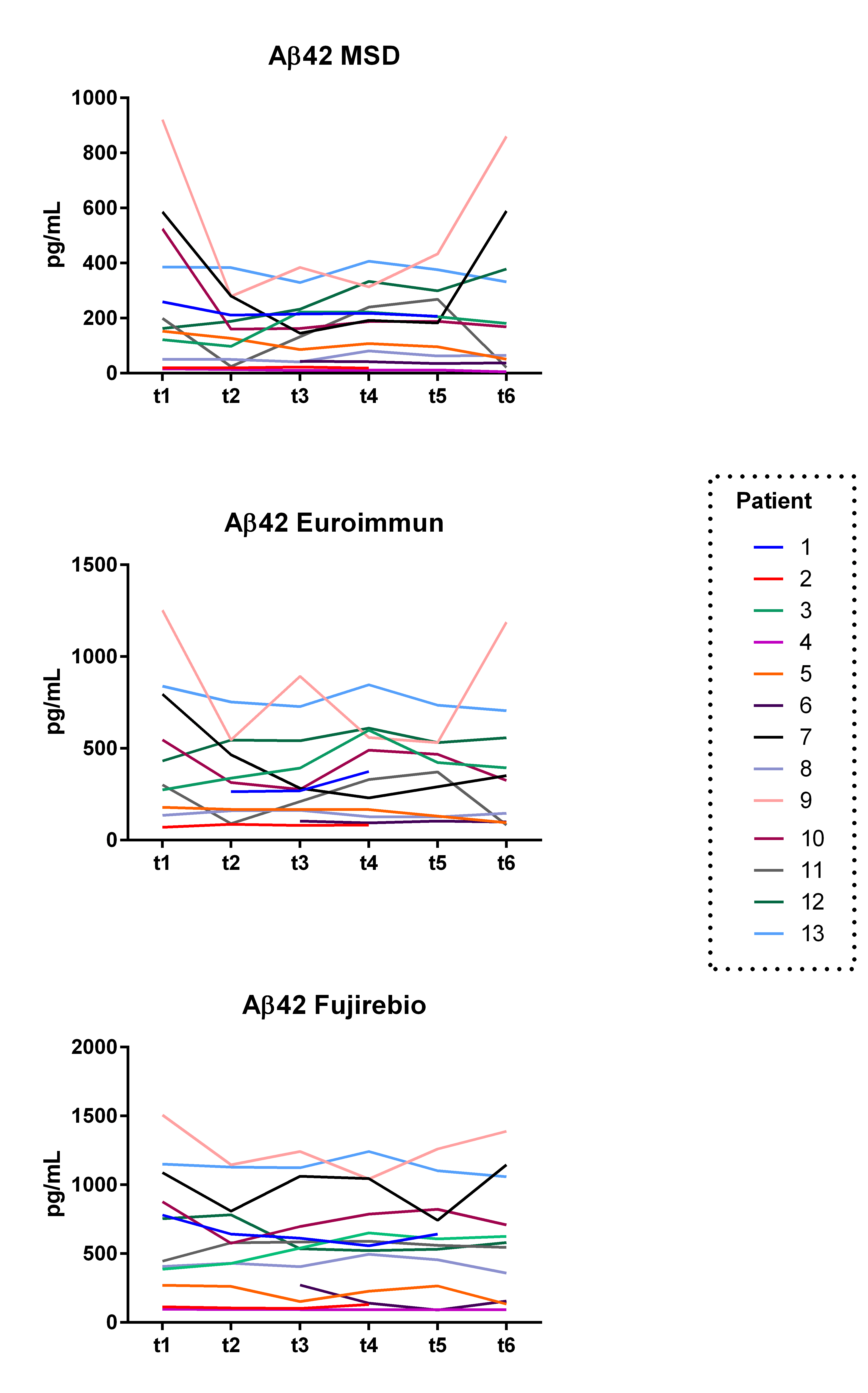

Supplement: Additional file 3: — Intraindividual concentration levels of Aβ42 across time points measured with different assays (Euroimmun, MSD, Fujirebio). (JPG 2223 kb) [file 13024_2016_130_MOESM3_ESM.jpg]
